# Dual-Polarized Multi-Channel 24 GHz Radar Sensor Antenna for High Channel-to-Channel Isolation

**DOI:** 10.3390/s20185233

**Published:** 2020-09-14

**Authors:** Young-Jun Kim, Gunhark Noh, Han Lim Lee, Sungwook Yu

**Affiliations:** School of Electrical and Electronics Engineering, Chung-Ang University, 84 Heukseok-ro, Dongjak-gu, Seoul 06974, Korea; yhjkim747@cau.ac.kr (Y.-J.K.); nogunhark23@cau.ac.kr (G.N.); hanlimlee@cau.ac.kr (H.L.L.)

**Keywords:** dual-polarization antenna, high isolation antenna, multi-beam radar sensor, multi-channel radar sensor, polarization diversity sensor

## Abstract

This article presents a dual-polarized, high gain multi-beam and high T/Rx channel-to-channel isolation antenna module for 24 GHz sensor applications. The proposed antenna is configured to support 2-Tx and 2-Rx channels with a pair of vertically polarized (VP) radiation pattern and a pair of horizontally polarized (HP) radiation pattern. Further, each linearly polarized T/Rx antenna is configured by 2 × 4 array with a multi-layer integrated feed network, resulting in four sets of 2 × 4 array antennas fabricated within a single printed circuit board (PCB). Since multiple RF channels must be ensured with minimal interference, high antenna-to-antenna, including Tx-to-Tx, Rx-to-Rx, and Tx-to-Rx port isolations in the proposed antenna are achieved by multi-layered feed network and four sets of T-shaped magnetic walls. To verify the performance of the proposed structure, a 2-Tx and 2-Rx antenna module was fabricated at 24 GHz. The fabricated antenna showed a measured maximum 10-dB impedance bandwidth of 3.9% with a maximum measured gain of 11.7 dBi, considering both Tx and Rx. Further, the measured channel-to-channel isolations were always better than 35.6 dB at 24 GHz.

## 1. Introduction

**Figure 1:**
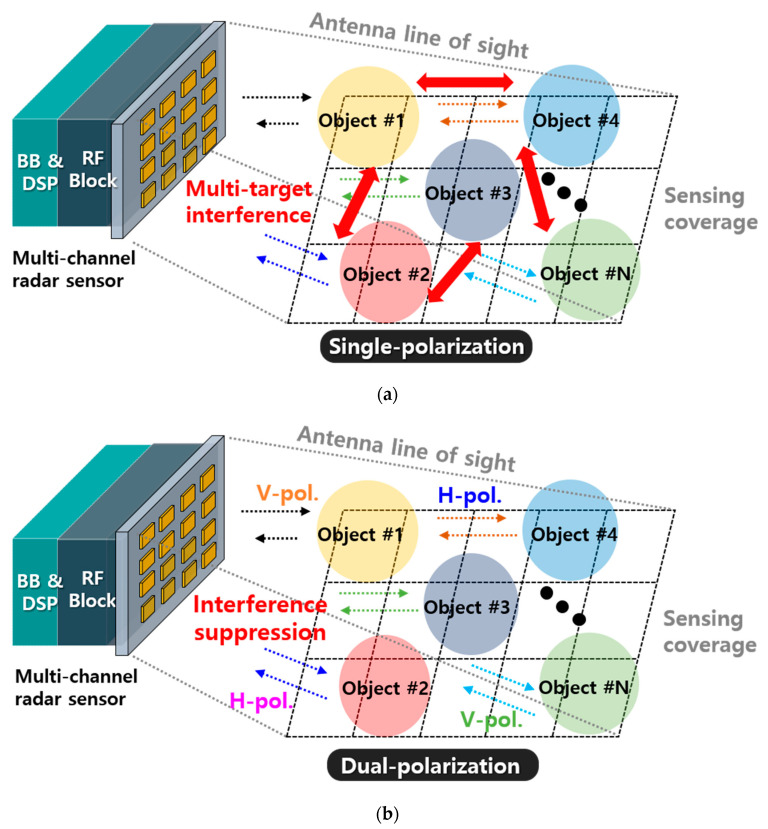
Multi-channel radar sensors (**a**) without having polarization diversity and (**b**) with a dual-polarization by VP and HP for interference suppression.

Sensors have become the key enabling technologies of the Internet-of-Things (IoT) applications such as manufacturing and industrial support [1,2,3,4,5], transportation and mobility [1,2,3,4,5,6], energy [7,8], retail [9,10,11], smart cities [12,13], health care [14,15], supply chain [16,17,18], agriculture [19,20], and buildings [21,22,23]. With these growing demand for contactless and wireless data acquisition and services, the demand for optimized sensors in various fields is also growing. To further extend the IoT potentials, recent research has focused on enhancing the data collection and analysis capabilities, and flexible sensor configuration within the IoT platform. Although the IoT sensor hierarchy includes many different levels such as raw data collection and analysis [24,25,26,27,28,29], motion detection [30,31,32], object analysis [33,34,35], activity monitoring [36,37,38,39], and context computation [40,41,42,43,44], the physical level of gathering raw data must be of primary concern. That is, as a massive number of wireless sensors carrying a massive amount of data at different frequencies, the antenna technologies capturing the required data at the desired frequencies must be ensured. Owing to increased sensor connectivity among multiple devices, an unavoidable level of microwave interference exists and thus potentially causes a significant degradation in the sensors’ performance [45,46,47,48]. To avoid performance degradation, the use of antennas with polarization diversity is considered to be one of the promising techniques to facilitate multiple microwave sensor operation with a reliable interference rejection [49,50,51,52,53]. There are typically four types of antenna polarizations, such as horizontal polarization (HP), vertical polarization (VP), right-hand circular polarization (RHCP), and left-hand polarization (LHCP). The best hardware configuration can be the support for all polarization types, but two polarization types can still be effective regarding circuit complexity, ease of integration, and cost for reconfigurable polarization RF-chain. Figure 1 shows the conceptual diagram for multi-channel radars with and without having polarization diversity. Typically, a radar sensor is capable of acquiring data such as target speed, target distance, and target location, whereas a multi-channel radar sensor must capture multiple target information simultaneously. As an example, Figure 1a shows multiple object identification or detection with a single polarization. Here, the multi-channel radar detects various information by simultaneously transmitting and receiving signals for different objects within the sensing coverage. However, each signal is easily corrupted by the interferences from neighboring objects reflecting multiple signals with the same kind of polarization, resulting in the collection of inaccurate raw data. On the other hand, if the multi-channel radar can transmit and receive signals through different polarizations, as shown in Figure 1b, the spectral channels can be isolated according to their polarization types, since a VP channel only responds with VP and vice versa. Thus, an accurate and reliable capturing of multiple raw data can be collected for multiple targets.

To provide polarization diversity at high frequencies, multiple antennas with different types of fixed polarizations can be effectively used due to the physically reduced antenna size [54,55,56,57,58]. However, these reported antennas require an extra artificial magnetic conductor (AMC) layer, impractical patch, or feed geometry that makes them unsuitable as high-frequency applications. More importantly, insufficient isolation characteristics are obtained. Further, array architectures are required to increase antenna directivity and sensing distance. Especially for multi-channel radar sensors, the channel-to-channel isolation, including both antenna array and feed network, must be high to avoid the strong transmitter (Tx) signals directly leaking or coupling to the receiver (Rx) chain, resulting in a poor signal-to-noise ratio (SNR) and degradation in sensing performance. Although the polarization diversity antennas are physically separated, multiple antenna arrays, including feed networks, are fabricated within the same printed circuit board (PCB) to form a single sensor module. Thus, although the electromagnetic radiation patterns for Tx and Rx are highly isolated, the conduction path isolation among each array corresponding to each channel cannot be sufficiently ensured. Although there has been previous research to ensure multi-channel isolation based on a dielectric slab [59], slotted-ground [60], SIW cavity [61], antenna spacing adjustment [62], SIW slot [63], and waveguide SIW [64], they suffer from either insufficient isolation, bulky cavity, or feed geometry, or incapability of simultaneous TRX operation. Therefore, in this article, a dual-polarized antenna module with high antenna gain and high channel-to-channel isolation is presented. The presented antenna module uses 24 GHz to operate in industrial, scientific, and medical (ISM) band, which is available without any restriction. Further, multiple antenna arrays with a reasonable size can be made in this band.

## 2. Proposed Antenna Design

**Figure 2:**
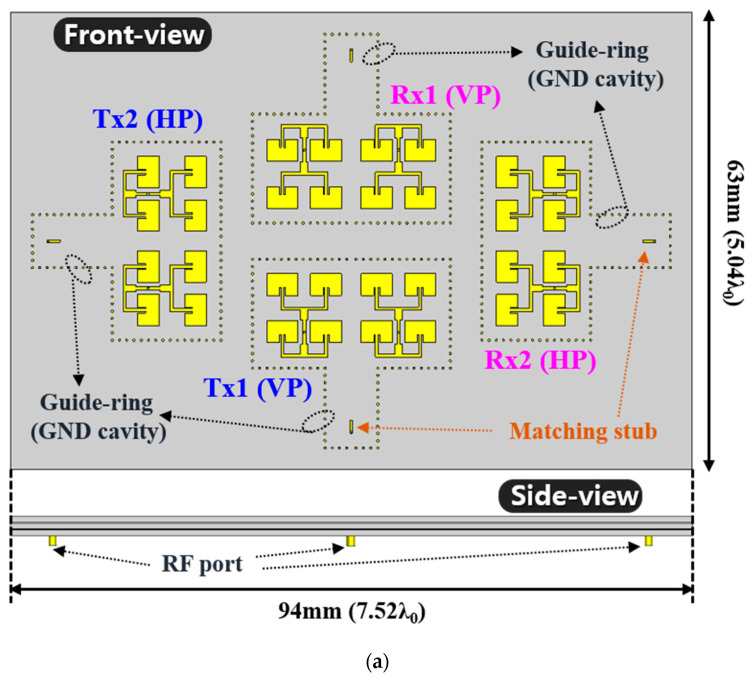

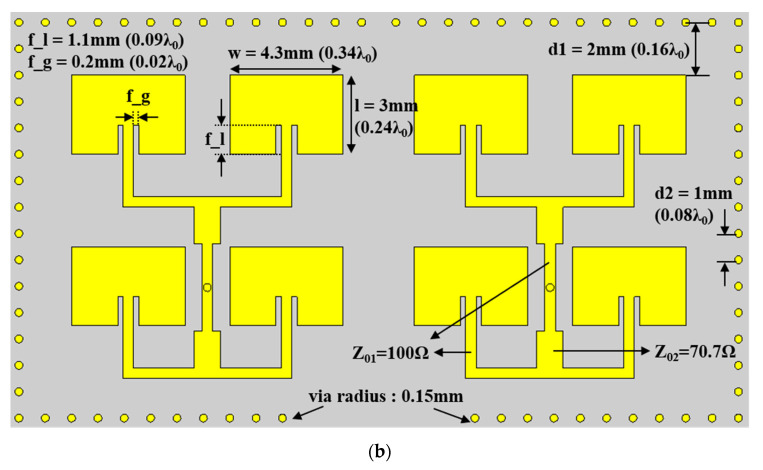
Proposed 2-Tx and 2-Rx radar sensor antenna module with dual-polarization: (**a**) overview and (**b**) detailed design parameters for one array with a guide-ring.

Figure 2 shows the detailed description of the proposed antenna structure with the front and side views as well as the detailed design parameters related to guide-rings. Each patch element is matched at 100 Ω and thus T-junction feed network with an impedance transformer is used.

**Figure 3:**
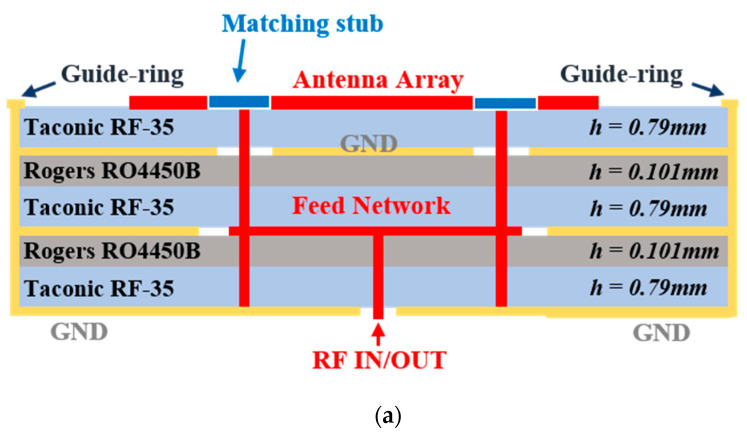

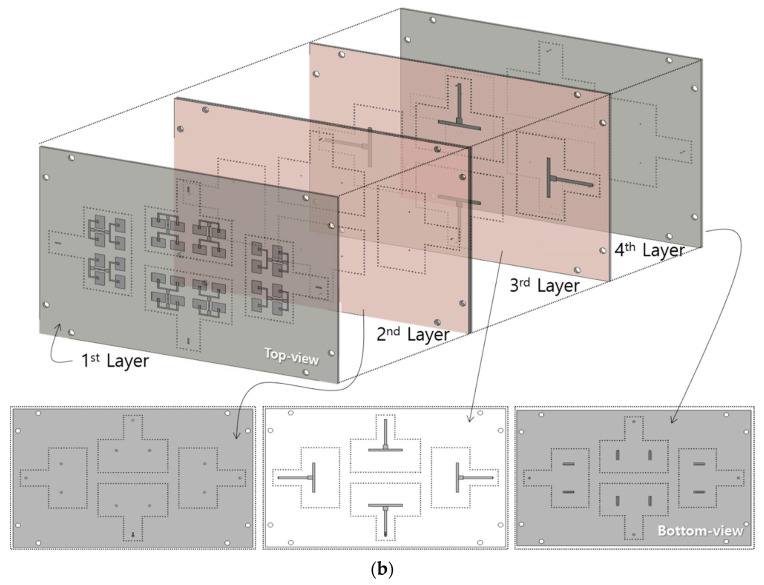
Proposed antenna configuration with the (**a**) multi-layer information and (**b**) detailed views for each layer.

The proposed antenna module was designed for a 2-Tx and 2-Rx radar sensor application with a pair of HP radiation and a pair of VP radiation patterns. Further, each antenna was identical and configured by a 2 × 4 array with an integrated feed network, a feed matching stub, and a guide-ring providing ground cavity for high antenna-to-antenna isolation. Since each antenna was used to transmit or receive an RF signal regarded as a channel information, high isolation among each antenna ensures high channel-to-channel isolation in a radar sensor platform. Figure 3 shows the multi-layer structure of the proposed antenna module, where 4-layer PCB was used. Here, the RF-35 Taconic substrate had a relative permittivity of 3.5 and loss tangent of 0.0018, whereas RO4450B was used as a prepreg. The radiation patch arrays and impedance matching stub for the feed network were designed on the upper layer. The second and third inner layers were used for the feed network and ground plane for the antenna elements. The bottom layer was used for the ground plane and RF ports. Further, a guide-ring for each array was implemented through the whole layers. That is, the proposed antenna structure adopted four guide-rings configured by multiple ground vias for each antenna array, including the feed network.

**Figure 4:**
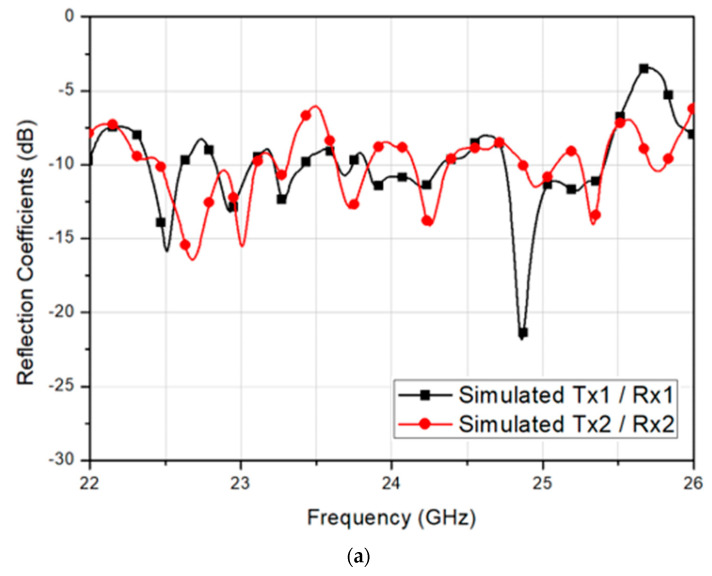

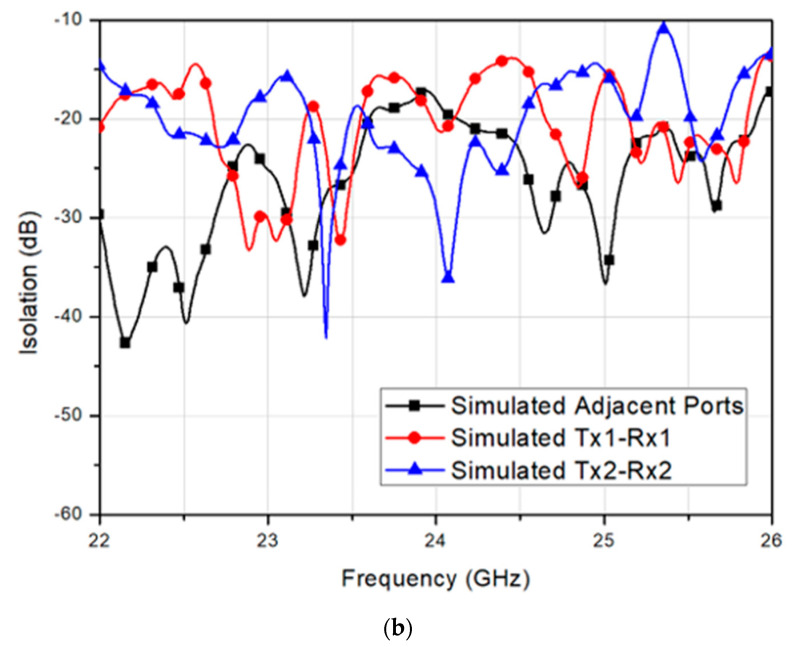
Simulated (**a**) reflection coefficient and (**b**) isolation of the 2-Tx and 2-Rx antenna module without the guide-rings.

Although the neighboring antennas can be isolated by polarization diversity, the four arrays within a single antenna module shared a relatively large common ground plane, resulting in a field distribution susceptible to the neighboring antenna condition. Firstly, the reflection coefficients and isolation characteristics of 2-Tx and 2-Rx arrays without including guide-rings simulated by using a 3-D EM simulator, CST Microwave Studio, and 2.92 mm RF connector model as shown in Figure 4.

**Figure 5:**
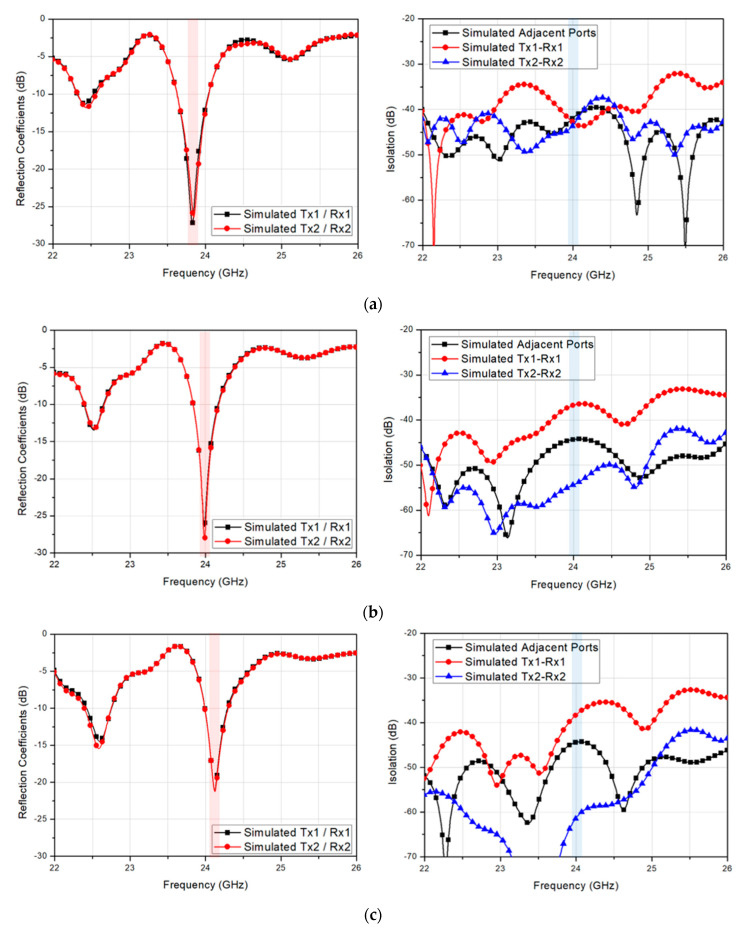
Simulated reflection coefficient and isolation of the 2-Tx and 2-Rx antenna with a guide-ring vias having a radius of (**a**) 0.1 mm (**b**) 0.15 mm and (**c**) 0.2 mm.

Since Tx1 and Tx2 are symmetric with Rx1 and Rx2, only two plots representing each symmetric pair are presented for better visibility in Figure 4a. It should be noted that the individual array has been matched at 24 GHz, but the integration of four arrays resulted in a deviation in reflection coefficients. The reflection coefficients were unstable, where each polarization antenna pair showed different return loss. The Tx2 and Rx2 antennas had the impedances unmatched at the center frequency, whereas Tx1 and Rx1 satisfied the matching at 24 GHz, as shown in Figure 4a. Further, it should be noted that since Tx1 and Tx2 were symmetric with Rx1 and Rx2, the symmetric coupling results other than Tx1-to-Rx1 and Tx2-to-Rx2 were denoted as adjacent ports, while identical plots were omitted for better visibility in Figure 4b. Without having the guide-rings, the simulated minimum isolation of 18 dB was observed between Rx1 and Rx2 antennas at 24 GHz. Thus, referring to the parameters described in Figure 2b, the guide-ring was added and optimized by using CST simulation. The distance between the patch and the guide-ring, d1, was determined by comparing the reflection coefficient tendency of the four-array to a single-array standalone case. Then, the simulated reflection coefficient and isolation with respect to the guide-ring via radius were achieved, as shown in Figure 5.

As previously mentioned, the redundant plots due to the antenna symmetry are omitted again in Figure 5. When the guide-ring via radius was set to 0.1 mm, the center frequency was observed at a slightly lower frequency than 24 GHz, as shown in Figure 5a. As the via radius increased, the center frequency shifted slightly higher, as shown in Figure 5b,c. The simulated minimum isolations at 24 GHz for via radius of 0.1 mm, 0.15 mm, and 0.2 mm were 41.9 dB, 36.7 dB, and 38.3 dB, respectively, whereas the maximum isolations were 43.5 dB, 54.4 dB, and 61.3 dB, respectively. Although the minimum isolation characteristic was shown the best with the via radius of 0.1 mm, the target frequency must be satisfied, and thus, the via radius was chosen as 0.15 mm.

**Figure 6:**
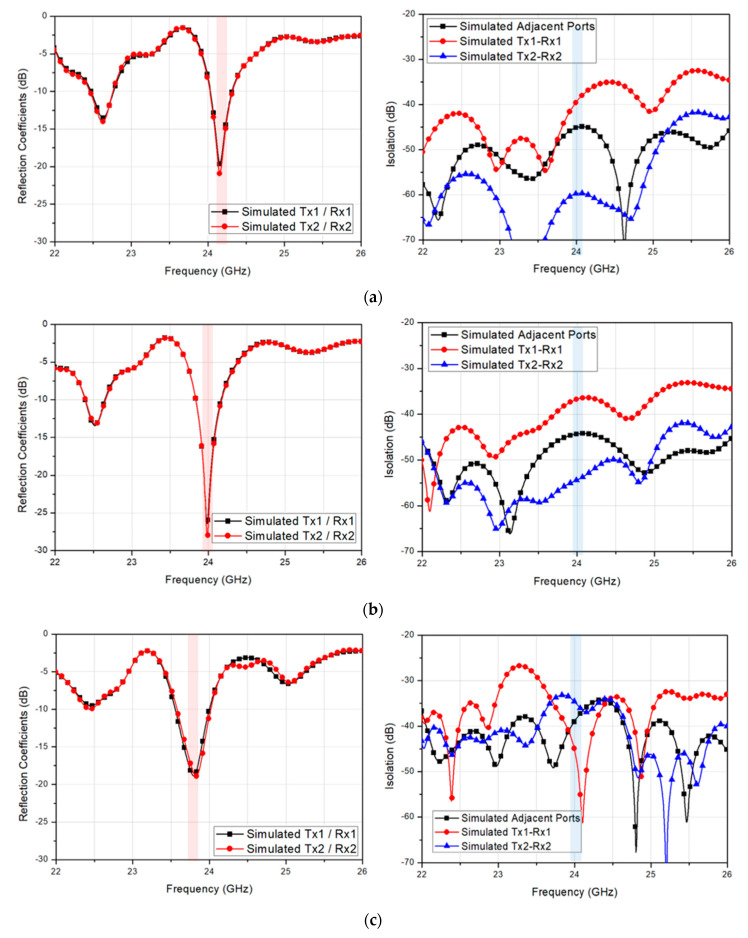
Simulated reflection coefficient and isolation of the 2-Tx and 2-Rx antenna with the guide-ring via having a separation distance of (**a**) 0.5 mm (**b**) 1.0 mm, and (**c**) 1.5 mm.

Thus, the effect of the distance between each via, d2, on the reflection coefficient and isolation were further simulated, as shown in Figure 6. When the guide-ring vias were placed with a distance of 0.5 mm, the center frequency was observed at a slightly higher frequency than 24 GHz, as shown in Figure 6a. As the via distance increases, the center frequency shifted slightly lower, as shown in Figure 6b,c. The simulated minimum isolations at 24 GHz for via distance of 0.5 mm, 1.0 mm, and 1.5 mm were 39.4 dB, 36.7 dB, and 34.7 dB, respectively, whereas the maximum isolations were 59.7 dB, 54.4 dB, and 45.5 dB, respectively. Although the minimum isolation characteristic was shown the best with the via separation distance of 0.5 mm, the target frequency must be satisfied, and thus, the via distance was chosen as 1.0 mm.

**Figure 7:**
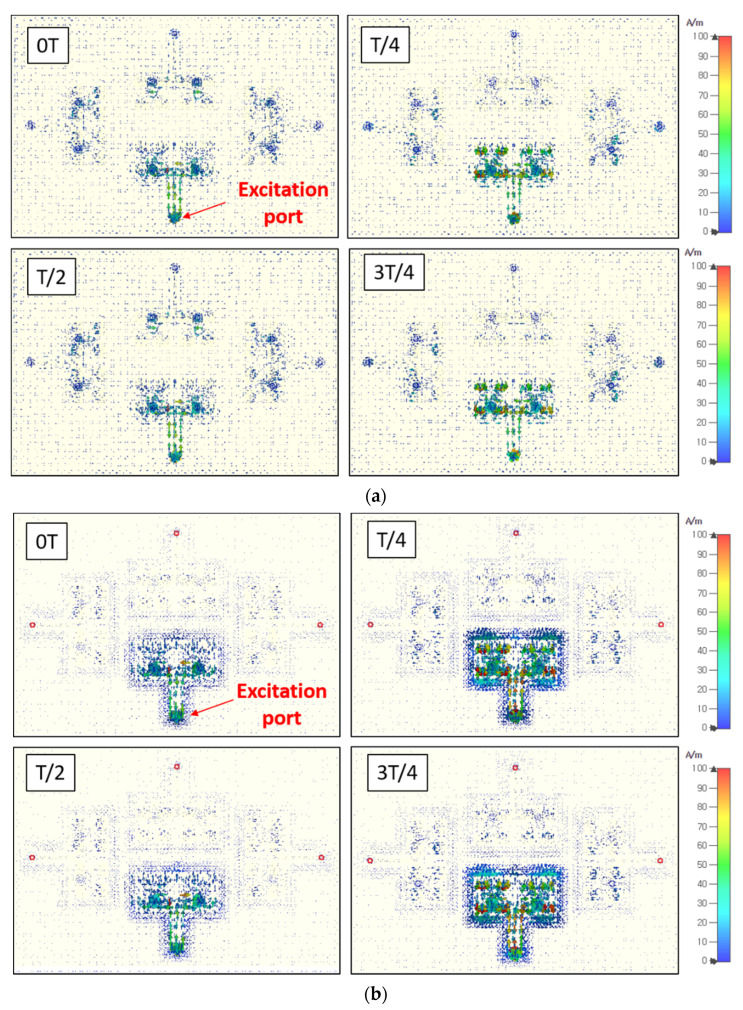
Simulated surface current distribution by a single port excitation for the antennas (**a**) without guide-rings and (**b**) with guide-rings at 24 GHz with different phases within a period.

To verify the isolation enhancement through the guide-ring effect, the surface current distribution at 24 GHz was simulated by a single port excitation with and without guide-ring vias at different phases within a period, as shown in Figure 7. Again, the simulation was conducted by CST, where hexahedral mesh at 26 GHz (for 22 GHz to 26 GHz band) with –25 dB accuracy was set.

Figure 7a shows the surface current spreading all over the ground plane and flowing to the neighboring antennas. These surface currents directly leaked to the other antennas and thereby caused the impedance instability and isolation degradation. To prevent the multi-channel antenna isolation from being degraded and unstable, the proposed structure adopted guide rings for each array. As shown in Figure 7b, the surface current was strongly held inside the guide ring, resulting in a stronger field excitation for the desired antenna array, whereas the unwanted surface current leaking to the neighboring antenna arrays is significantly reduced. Thus, higher antenna port-to-port isolation characteristic among multiple antenna arrays can be achieved compared to the simple combination of orthogonal antenna placement.

**Figure 8:**
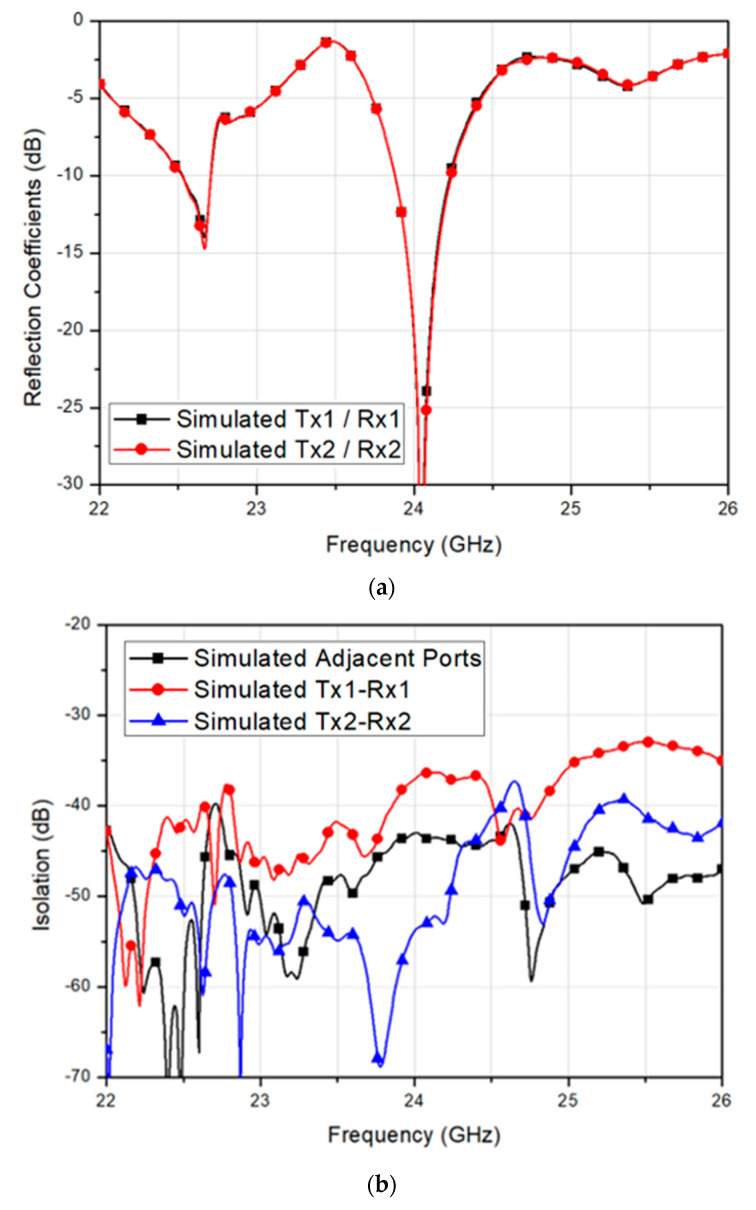
Simulated (**a**) reflection coefficient and (**b**) isolation magnitude of the 2-Tx and 2-Rx antenna module with the guide-rings and feed network impedance matching stubs.

However, by simply having the guide-ring cannot guarantee the antenna performance because the area for the surface current that can be circulated gets changed by the magnetic walls. That is, the antenna impedance for each array should be optimized by taking the guide-ring into account. Thus, the proposed feed network adopted an integrated feed network connected with a vertical matching stub on the top-layer, as described in Figure 3. Then, to verify the S-parameters of the proposed antenna module, the reflection coefficient and isolation for the 2-TX and 2-Rx with the guide-rings and matching stubs were simulated, as shown in Figure 8.

**Figure 9:**
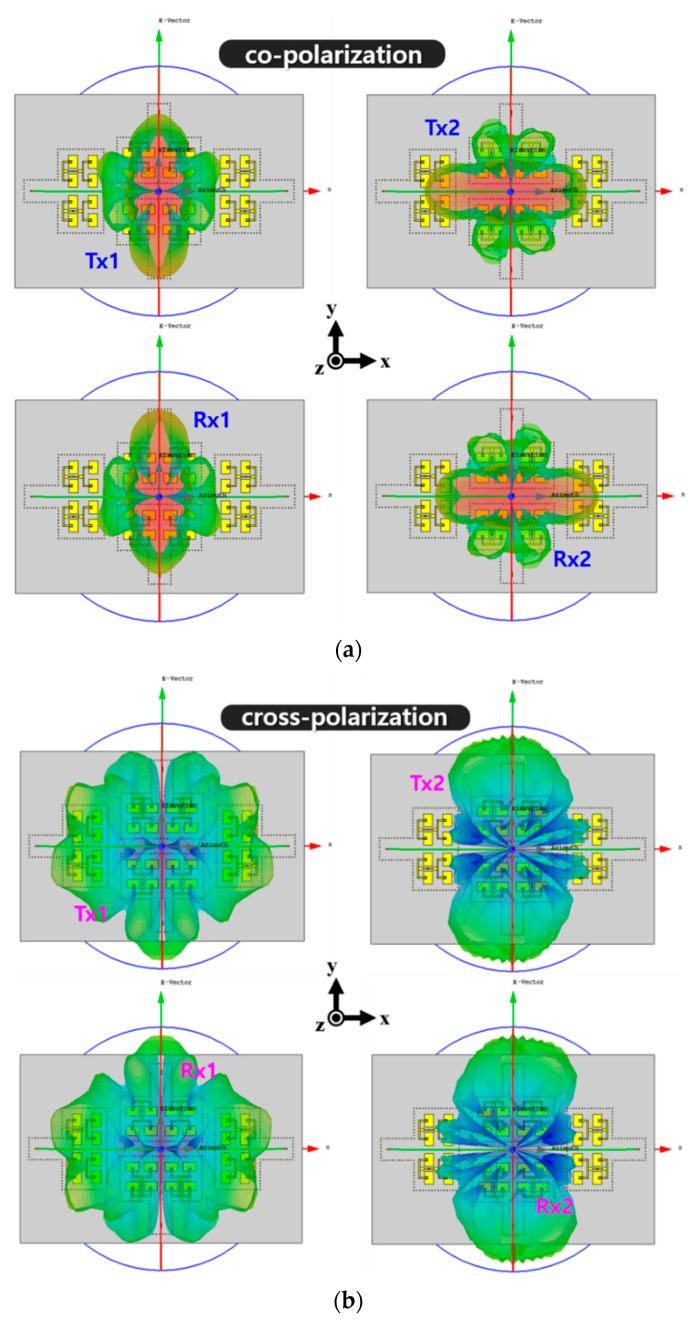
Simulated 3-D radiation patterns of the proposed antenna module with (**a**) co-polarization patterns and (**b**) cross-polarization patterns for each channel.

All antennas with the guide-rings showed identical reflection coefficients with the center frequency at 24 GHz, as shown in Figure 8a. The simulated 10-dB impedance bandwidth of the proposed antenna is 357 MHZ corresponded to a fractional bandwidth of 1.49%. Also, the simulated minimum isolation of 37.1 dB was observed between Tx1 and Rx1 at 24 GHz, resulting in the minimum isolation enhanced by 206% approximately, as shown in Figure 8b. Further, the maximum isolation level is about 53.9 dB and achieved by Tx2-to-Rx2 path. Then, the 3-D radiation patterns for co-polarization and cross-polarization characteristics of the proposed 2-Tx and 2-Rx antenna module was simulated, as shown in Figure 9.

**Figure 10:**
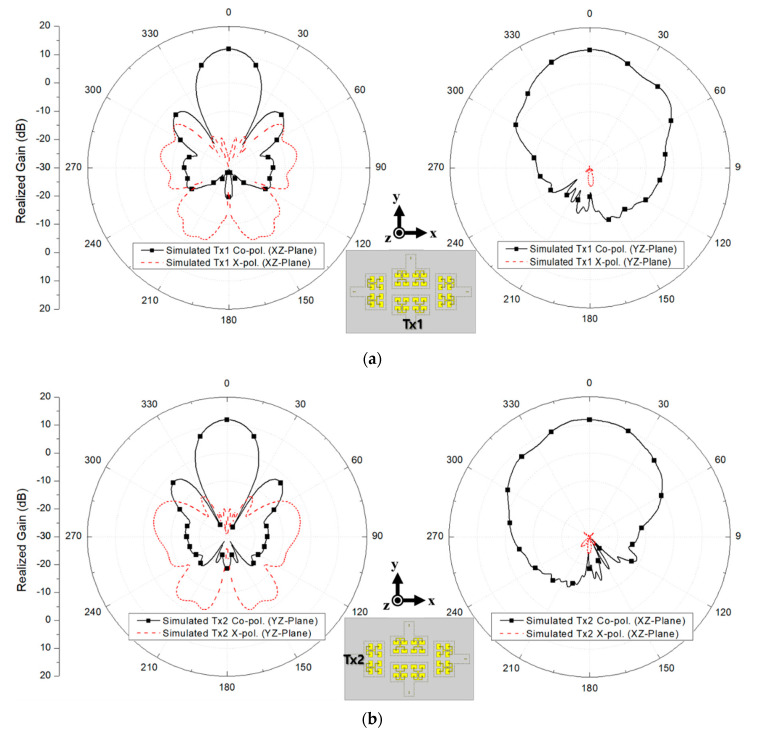

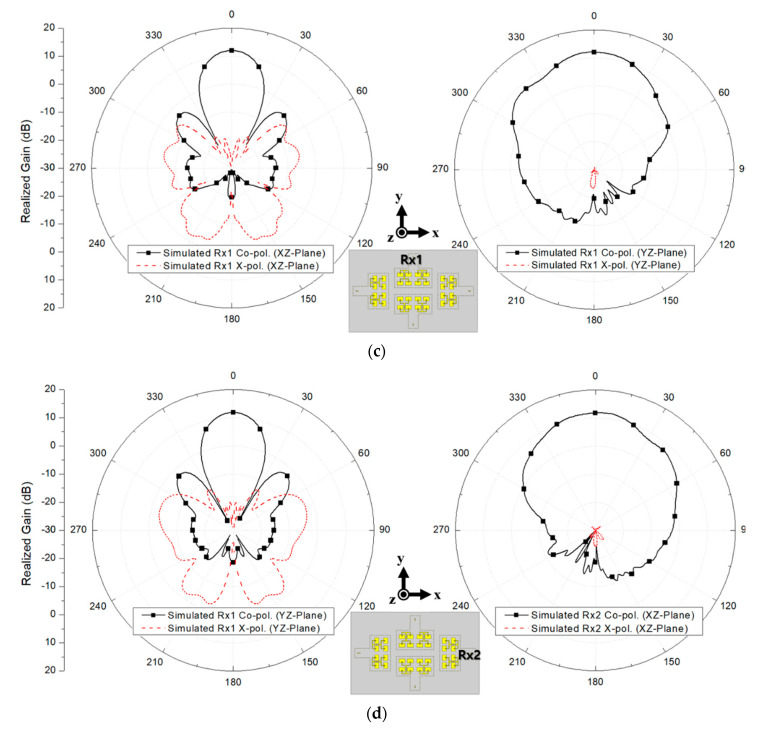
Simulated radiation patterns for (**a**) Tx1, (**b**) Tx2, (**c**) Rx1 and (**d**) Rx2 in xz and yz planes.

To clarify the radiation characteristics, the polar patterns for each array in the corresponding radiation planes are presented in Figure 10. The simulated peak gain of Tx1, Tx2, Rx1, and Rx2 were 12.0 dBi, 11.9 dBi, 12.0 dBi, and 11.9 dBi, respectively. The simulated half-power beamwidth (HPBW) for Tx1, Tx2, Rx1, and Rx2 in xz plane were 24°, 49°, 24°, and 49°, respectively, while the simulated HPBW in yz plane for Tx1, Tx2, Rx1, and Rx2 were and 46°, 24°, 42° and 24°, respectively. Further, the simulated 0-dB beamwidth for Tx1, Tx2, Rx1, and Rx2 in xz plane were 48°, 122°, 48°, and 122°, respectively. Lastly, the simulated 0-dB beamwidth for Tx1, Tx2, Rx1, and Rx2 in yz plane were 129°, 46°, 130°, and 46°, respectively. It should be noted that the simulated cross-polarizations of Tx1 and Rx1 in yz plane and Tx2 and Rx2 in xz plane were normalized by −40 dB in Figure 10 since the levels were extremely small. These simulated radiation patterns will be also compared with the measured results in the next section.

## 3. Measured Results

**Figure 11:**
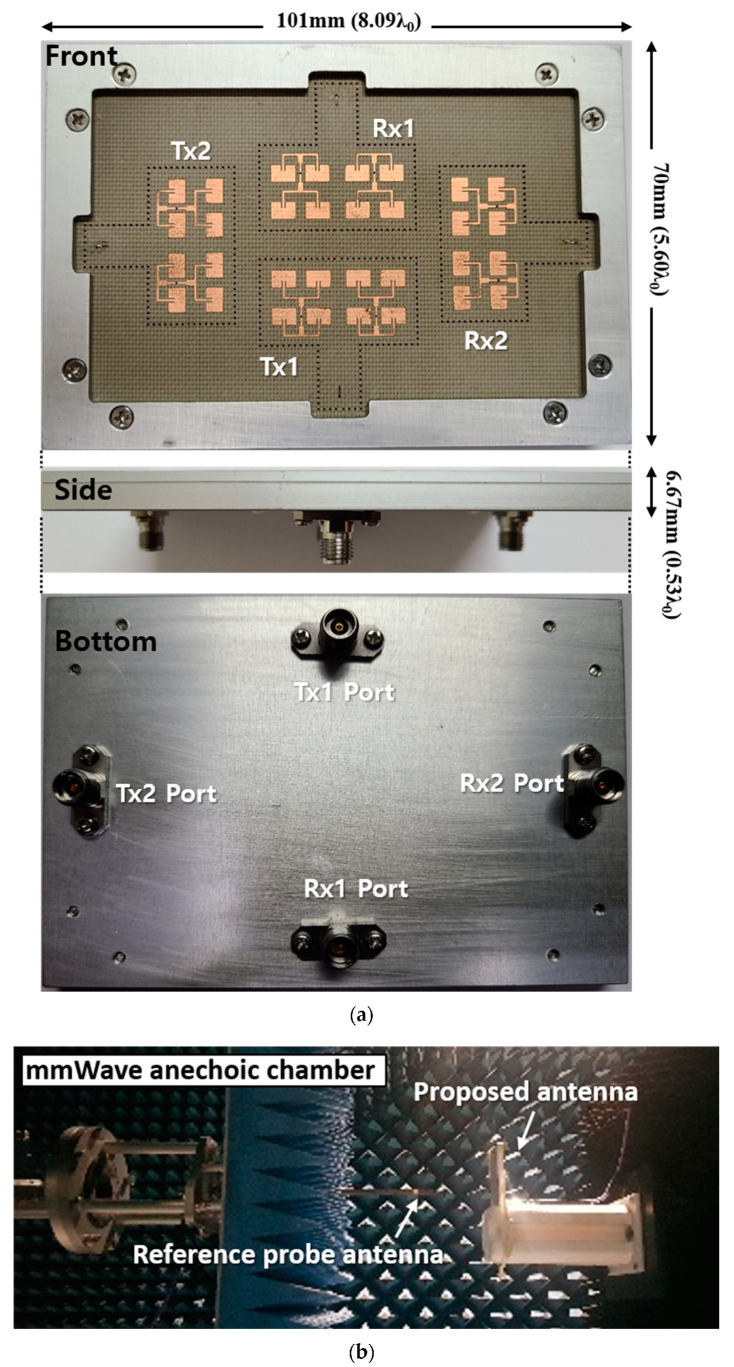
The proposed 2-Tx and 2-Rx antenna module: (**a**) fabrication photo and (**b**) measurement setup at mmWave anechoic chamber.

The proposed 2-Tx and 2-Rx antenna module was fabricated with the 4-layer PCB configured by RF-35 Taconic substrates having a relative permittivity of 3.5 and loss tangent of 0.0018, and RO4450B prepregs having a relative permittivity of 3.35 and loss tangent of 0.004, as shown in Figure 11a. The detailed layer information can be referred to in Figure 3b in the previous section. The fabricated dimension of the proposed antenna module, including the test jig and excluding the RF connectors, was 8.09 λ_0_ x 5.60 λ_0_ x 0.53 λ_0_ at 24 GHz. It is noted that the actual antenna PCB occupation, including the ground plane, was 7.52 λ_0_ x 5.04 λ_0_. Then, the S-parameters were measured by Keysight N5227B PNA vector network analyzer, and the radiation patterns of the proposed antenna were measured with a near-field scanning at a mmWave anechoic antenna chamber, as shown in Figure 11b.

**Figure 12:**
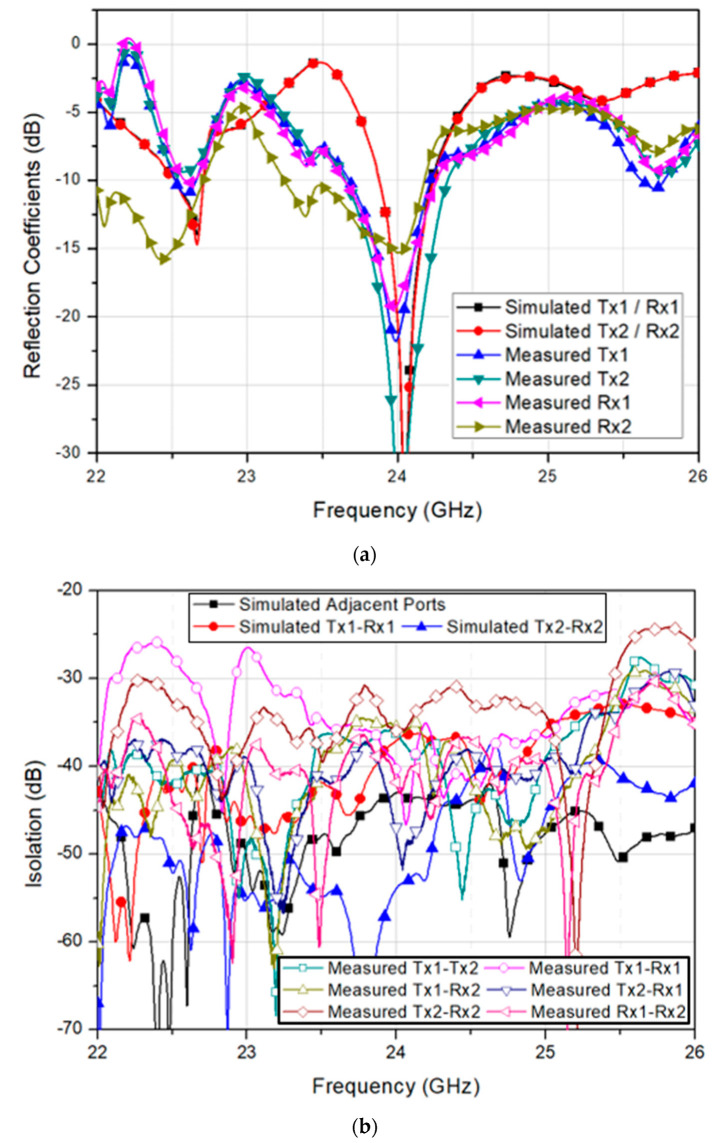
Simulated and measured (**a**) reflection coefficients and (**b**) isolation characteristics of the fabricated 2-Tx and 2-Rx antenna module.

Figure 12a shows the measured reflection coefficient of the fabricated 2-Tx and 2-Rx antenna module in comparison with the simulated results. The measured 10-dB impedance bandwidths of the Tx1, Tx2, Rx1, and Rx2 were 552 MHz, 679 MHz, 679 MHz, and 931 MHz, resulting in fractional bandwidths of 2.3%, 2.8%, 2.8%, and 3.9%, respectively. The measured bandwidths were a little wider than the simulated bandwidths due to the effect of test jig and real RF connectors. Nevertheless, the measured reflection coefficient showed a good agreement with the simulated result.

**Figure 13:**
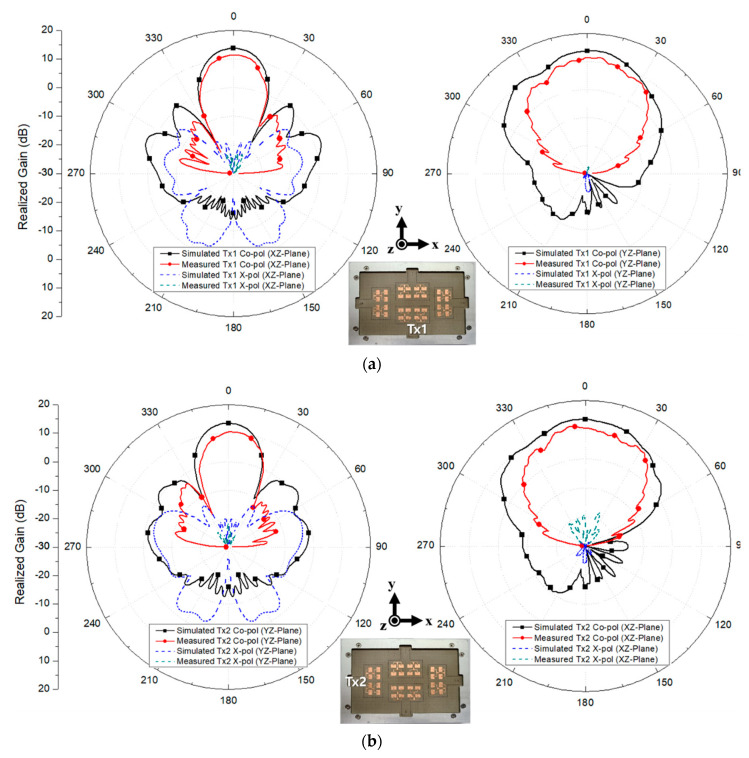

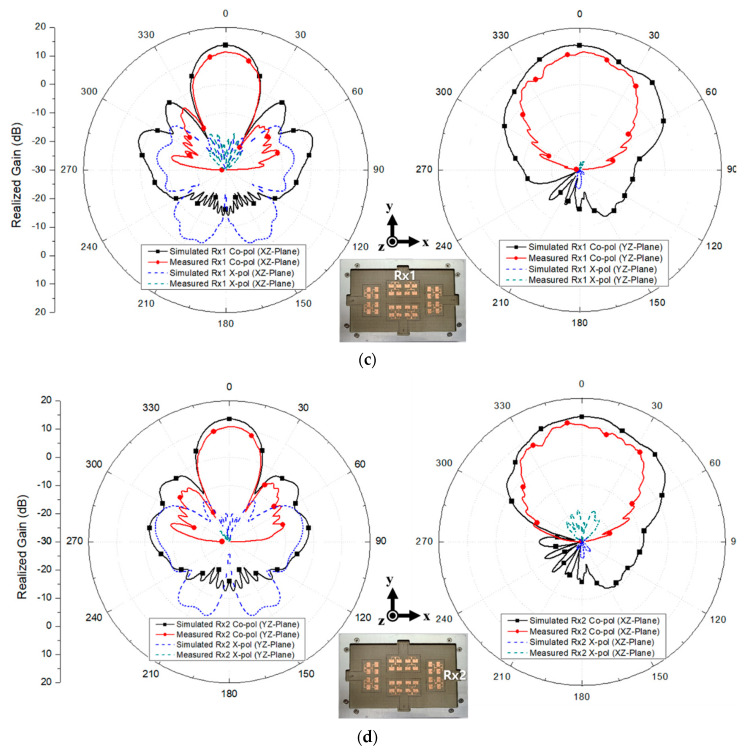
Measured radiation patterns for (**a**) Tx1, (**b**) Tx2, (**c**) Rx1, and (**d**) Rx2 in xz and yz planes.

Further, Figure 12b shows the measured isolation characteristics among Tx and Rx arrays in comparison with the simulated results. The minimum measured isolation was found by 35.6 dB between Tx1 and Rx2, whereas the maximum measured isolation of 47.0 dB was achieved by Tx2-to-Rx1 path. Therefore, both measured and simulated isolation characteristics showed high isolation among Tx and Rx arrays, and a good agreement. Once the measured S-parameters had been verified by showing good performances, the radiation patterns were also measured at an anechoic antenna chamber and compared with the simulated results at 24 GHz. The measurement results are shown in Figure 13.

The measured peak gain for Tx1, Tx2, Rx1, and Rx2 were 11.7 dBi, 11.4 dBi, 11.6 dBi, and 11.6 dBi, respectively. The slight decrease in the realized gain compared to the simulated gain was due to the RF connector loss. Further, the measured HPBW in xz plane for Tx1, Tx2, Rx1, and Rx2 were 24°, 35°, 23° and 36°, respectively, whereas the measured HPBW in yz plane for Tx1, Tx2, Rx1, and Rx2 were 37°, 21°, 34° and 21°, respectively. The measured 0-dB beamwidths for Tx1, Tx2, Rx1, and Rx2 in xz plane were 45°, 89°, 46°, and 88°, respectively. Lastly, the measured 0-dB beamwidths for Tx1, Tx2, Rx1, and Rx2 in yz plane were 89°, 44°, 84°, and 44°, respectively. Although the measured beamwidths were smaller than the simulated beamwidths due to the inclusion of the test jig, the measurement showed a reasonable range of agreement with the simulation regarding the performance tendency. The measured results are summarized in Table 1 and verify the high channel-to-channel isolation with good radiation characteristics at 24 GHz applications.

Finally, the performance comparison of the proposed antenna module with the other antennas is summarized in Table 2, showing the excellent isolation characteristic of the proposed structure.

## 4. Conclusions

The dual-polarized, high gain multi-beam, and high T/Rx channel-to-channel isolation antenna module for 24 GHz applications have been presented. The proposed antenna module consisted of 2-TX and 2-Rx, where each antenna was configured by 2 × 4 array with a guide-ring and a matching stub to ensure high isolation and stabilized matching performances. The proposed antenna module was fabricated by 4-layer PCB process and showed a good agreement between the simulated and measured results. The measured 10-dB impedance bandwidths for Tx1, Tx2, Rx1, and Rx2 were 2.3%, 2.8%, 2.8%, and 3.9%, respectively, while the measured minimum isolation among the arrays was suppressed below 35.6 dB at 24 GHz. Moreover, the highest isolation achieved at 24 GHz was about 47 dB. Further, the measured peak gains for Tx1, Tx2, Rx1, and Rx2 were 11.7 dBi, 11.4 dBi, 11.6 dBi, and 11.6 dBi, respectively, including the feed loss. The measured HPBW in xz plane for Tx1, Tx2, Rx1, and Rx2 were 24°, 35°, 23° and 36°, respectively, whereas the measured HPBW in yz plane for Tx1, Tx2, Rx1, and Rx2 were 37°, 21°, 34° and 21°, respectively. Lastly, the measured 0-dB beamwidths for Tx1, Tx2, Rx1, and Rx2 in xz plane were 45°, 89°, 46°, and 88°, respectively, whereas the measured 0-dB beamwidths for Tx1, Tx2, Rx1, and Rx2 in yz plane were 89°, 44°, 84°, and 44°, respectively. Thus, the proposed antenna module can be efficiently used for multi-channel radar sensor applications such as smart IoT-based environments where strong Tx leakages to Rx chain can be greatly suppressed, resulting in an enhanced dynamic range for both Tx and Rx operation.

## Figures and Tables

**Table 1:** Summary of the measured performance for the proposed 2-Tx and 2-Rx antenna module.

Antenna Array	10-dB BW (%)	Realized Gain (dBi)	E-Plane HP/0-dB BW (°)	H-Plane HP/0-dB BW (°)	Min. Isolation (dB)
Tx1	2.3	11.7	24/45	37/89	36.2 (Tx2)
Tx2	2.8	11.4	35/89	21/44	35.6 (Rx1)
Rx1	2.8	11.6	23/46	34/84	35.6 (Tx2)
Rx2	3.9	11.6	36/88	21/44	36.0 (Tx2)

**Table 2:** Performance comparison of the proposed antenna module with various topologies.

Ref.	Isolation Topology	# of T/Rx Antennas *	Polarization/Diversity	Frequency (GHz) **	BW (%)	Realized Gain (dBi)	Min. Isolation (dB)
[59]	Dielectric slab	4–Tx (Rx)	CP (Dual)	37.5	29.3	12.8	15
[60]	Ground slot	4-Tx (Rx)	LP (Dual)	34.5	5.3	19.2	25
[61]	SIW cavity	4-Tx (Rx)	LP (Dual)	37	1.43	10.8	30
[62]	Antenna spacing	4-Tx (Rx)	RHCP (-)	28	6	9	24
[63]	SIW slot	16-Tx (Rx)	VP (-)	25.8	4.8	22.4	27
[64]	Waveguide SIW	16-Tx (Rx)	LP (Dual)	34.7	4.1	17.2	35
This-work	Guide-Ring	2-Tx & 2-Rx	LP (Dual)	24	3.9	11.7	35.6

* Tx (Rx) denotes the antennas that can be used only for Tx or only for Rx, or not specified. ** Highest operation frequency was chosen for those with multi-band operation.
